# “Invisible” visual impairments. A qualitative study of stroke survivors` experience of vision symptoms, health services and impact of visual impairments

**DOI:** 10.1186/s12913-020-05176-8

**Published:** 2020-04-15

**Authors:** Helle K. Falkenberg, Torgeir S. Mathisen, Heidi Ormstad, Grethe Eilertsen

**Affiliations:** 1 National Centre for Optics, Vision and Eye Care, Faculty of Health and Social Sciences, University of South-Eastern Norway, Hasbergs vei 36, 3616 Kongsberg, Norway; 2 USN Research Group of Older Peoples` Health, University of South-Eastern Norway, Drammen, Norway; 3 Department of Nursing and Health Science, Faculty of Health and Social Sciences, University of South-Eastern Norway, Drammen, Norway

**Keywords:** Rehabilitation, Qualitative research, Municipal- and specialist health care services, Health care professionals, Vision

## Abstract

**Background:**

Visual impairments (VIs) have a negative impact on life and affect up to 60% of stroke survivors. Despite this, VIs are often overlooked. This paper explores how persons with VIs experience vision care within stroke health services and how VIs impact everyday life the first 3 months post stroke.

**Methods:**

Individual semi-structured interviews were conducted with 10 stroke survivors 3 months post stroke, and analyzed using qualitative content analysis.

**Results:**

The main theme, “Invisible” visual impairments, represents how participants experience VIs as an unknown and difficult symptom of stroke and that the lack of attention and appropriate visual care leads to uncertainty about the future. VIs were highlighted as a main factor hindering the participants living life as before. The lack of acknowledgement, information, and systematic vision rehabilitation leads to feelings of being unsupported in the process of coping with VIs.

**Conclusion:**

VIs are unknown symptoms pre stroke and sequelas after stroke that significantly affect everyday life. VIs and vision rehabilitation needs more attention through all phases of stroke health services. We request a greater awareness of VIs as a presenting symptom of stroke, and that visual symptoms should be included in stroke awareness campaigns. Further, we suggest increased competence and standardized evidence-based clinical pathways for VIs to advance all stroke health services including rehabilitation in order to improve outcomes and adaptation to future life for stroke survivors with VIs.

## Background

Stroke is the second-leading cause of death [1], disability, and need for long-term rehabilitation and care in industrialized countries [2], and approximately 10.3 million persons suffer from stroke every year [3]. With an estimated increase of 60–80% in stroke events by 2050 [4], this will cause considerable challenges to the health care system, as half of those who survive will experience permanent complications or changes [1]. Impaired vision is one of many complications after stroke and affects up to 60% of stroke survivors [5].

Visual impairments (VIs) after stroke include visual field loss, eye movement disorders, loss of central vision and perceptual disorders. Symptoms of VIs include sudden loss of vision, double vision, reading difficulties, reduced balance or mobility, clumsiness or inattention of visual information [6, 7]. However, many stroke survivors do not relate these symptoms to impaired vision but attribute them to other problems, such as age, fatigue, and limb weakness [6]. VIs may be permanent and are associated with fatigue, mental distress, increased falls, and reduced rehabilitation outcomes and daily living activities [8–12]. They may lead to driving cessation [13], reading difficulties [14, 15], and increased falling [16]. Failing to identify VIs after stroke can have a severe negative impact on the patient’s coping, further recovery, and quality of life [17].

Learning to live with a visual loss is a comprehensive and complex task closely intertwined with existential and social aspects [18–20]. Regardless of severity, much can be done to recover from or improve VIs through vision rehabilitation [21, 22], even if high quality randomized controlled trial (RCT) studies do not show evidence of effective treatment for visual field loss [23]. Several studies have concluded that identifying and give advice on VIs and coping strategies will raise self-awareness [22], and that visual compensatory training is useful for vision outcomes [23, 24], rehabilitation of other consequences of stroke, and the overall rehabilitation process [25]. The Norwegian guidelines for treatment and rehabilitation of stroke [26] state that vision function should be examined after stroke, VI patients should be referred to an eye care specialist, and compensatory training is recommended after vision field loss [26]. In Norway, general stroke treatment and outcomes are successful [27]; the median hospital stay is 5 days, and 44% are discharged straight home [28]. However, in Norway and internationally, vision rehabilitation is not generally included in stroke health services and VIs are commonly overlooked and undertreated as professionals in both specialist and municipal health care services often have little awareness and competence regarding visual problems occurring in stroke survivors [6, 11, 19, 29–32]. That VIs are inadequately documented and lack systematic assessment, treatment, or rehabilitation [31, 33], leads to negative consequences not only for the individual but also for the family and society [19, 34–36].

In order to improve health services and outcomes for stroke survivors with VIs, a deeper understanding is needed of how stroke survivors view the way specialist and municipal health care services address and attend to VIs in stroke care, and further, how VIs affect everyday life in the first months after stroke. Today this knowledge is scarce. Further, this is the first Norwegian study illuminating patient perspectives regarding VIs and stroke health services early after stroke. Key aspects include symptoms, experiences of health services and the impact of VIs following stroke.

## Methods

### Study design

This qualitative study used a descriptive, interpretative design with in-depth individual participant interviews. Qualitative interviews aim to gain knowledge about the experiences of participants in relation to a particular phenomenon. Here, the phenomenon to be studied was stroke survivors` experiences of vision symptoms, health services and impact of VIs. The study is part of a larger knowledge translation project [37] that aims to implement knowledge and competence of vision care and rehabilitation in Norwegian stroke health services.

### Recruitment and participants

Stroke nurses in two acute stroke units recruited patients with VIs after stroke. All patients identified with VIs and who fulfilled the inclusion criteria were invited to participate during an eight-month period. Inclusion criteria were persons with VIs following acute stroke, over 18 years of age, the ability to express their experiences in Norwegian, and the ability to participate in an in-depth interview lasting approximately 60 min. VIs were identified by use of the National Institute of Health Stroke Scale (NIHSS) [38]. NIHSS is a standard test used on all patients with suspected stroke in the two hospitals. It includes a simple clinical assessment of visual field, horizontal eye movements and neglect. In addition, patients that reported double vision were asked for participation.

Five men and five women with a mean age of 73.4 years participated (see Table 1). Six were discharged directly home from the stroke units, while four had extensive rehabilitation due to physical and cognitive consequences following their stroke. All lived in their own homes at the time of the interview. Only one patient had received visual rehabilitation after self-referral. All participants stated that, before the stroke, they could see well and did not experience any problems with their vision. Everyone used reading glasses, most had undergone cataract surgery. The required sample size was assessed during data collection in order to ensure that sufficient data was obtained. The interviews provided rich and varied descriptions, and the sample of ten was considered appropriate.

**Table 1:** Participants’ characteristics

ID	Age	Gender	Days in acute care	Rehabilitation	Self-reported visual impairment
1	70	Female	14	In-patient	Hemianopia
2	59	Male	2	None	Loss of vision one eye
3	76	Female	21	In-patient	Hemianopia
4	75	Male	14	In-patient	Hemianopia/neglect
5	73	Male	14	None	Hemianopia
6	71	Female	7	In-patient	Hemianopia/neglect
7	81	Male	2	None	Hemianopia
8	71	Female	5–9 (unsure)	None	Hemianopia
9	90	Female	5	None	Diplopia
10	68	Male	4	None	Hemianopia

### Data collection—qualitative interviews

Three months after discharge from the acute stroke unit, a qualitative in-depth, semi structured interview was conducted with each of the participants. A research assistant conducted the interviews in the participants’ homes. An interview guide was developed focusing on the person’s experiences of the initial symptoms of stroke; vision care from the first point of contact, during the stay on the stroke unit, and in rehabilitation or municipal health care services. Further, details of living with VIs from stroke onset and during the following 3 months constituted a particular focus. The participants were encouraged to express themselves freely and elaborate if new topics emerged. All interviews were digitally recorded and transcribed verbatim.

### Data analysis

We performed a content analysis as described by Graneheim and Lundman [39, 40] in which both manifest and latent content were identified. Manifest content represents the obvious content—what the text says. Latent content represents the underlying meaning of what the text talks about and contains a higher level of interpretation and abstraction than the manifest content [40]. The analysis started with all authors reading all interviews as a whole text to get familiar with the content. Meaning units were then identified by TSM and HKF and discussed with HO and GE, and could be one word or a whole paragraph that expressed a specific meaning of interest to the research questions. The next step condensed the meaning units while still preserving the original meaning. Further, the condensed meaning units were abstracted and labelled with a code taking the context of the text into consideration [39, 40] (performed by TSM and HKF). All codes were discussed with all authors until consensus. Based upon similarities and differences, we grouped and abstracted the codes into seven categories. Table 2 shows an example of the analysis process from meaning unit (manifest content) to a category (latent content) [39, 40]. Further analysis and interpretation of the seven categories identified three subthemes. Finally, all authors agreed on one main theme after taking the original text, categories and subthemes into consideration, see Table 3.

**Table 2:** Examples of the analysis from manifest to latent content

Meaning unit (MU) Manifest content	Condensed MU Manifest content	Code Manifest content	Category Latent content
“I rubbed my eyes … but I thought it [visual loss] would pass—I did not think it could be something serious! But this was in the evening, so I took my medication, because I got tired, so I took them and went to bed. I fell asleep at once.”	Thought the vision loss would pass, and not as something serious.	Waiting for the symptoms to pass	Experience of sudden vision problems – distressful but not alarming
“I also lost balance, you know. It was not only that I did not see-that I saw double, but I was swaying. Just like a drunk man; I was swaying. That was unsettling. Very distressful.”	In addition to double vision, lost balance and was swaying. Felt like a drunk man swaying. Felt unsettled and very distressful.	Distressful feeling of sudden vison problem

**Table 3:** Presentation of categories, subthemes, and the main theme

Categories	Subthemes	Theme
• Experience of sudden vision problems – distressful but not alarming	Vision problems are experienced as a difficult unknown symptom of stroke	“Invisible” visual impairments
• Difficulties relating vision problems as a symptom of stroke		
• “They primarily do not have it in their checklist” - an experienced lack of focus on vision in the stroke units	Experiences of inadequate visual care in health services	
• No offer of visual rehabilitation in health services - a worry		
• Difficult and exhausting to adapt to changes in everyday activities	Visual impairments—big impact now and in the future	
• Life moves on, without driving		
• Being told that nothing could be done for the vision impairment - accepted, but not convinced …		

## Results

Through the analysis of interviewing ten stroke survivors of their experience of vision care after stroke and living with VIs the main theme, “Invisible” vision impairments, were derived from three subthemes and seven categories, and will be presented in this order, (see Table 3).

### “Invisible” vision impairments

The theme “Invisible” vision impairments covers both how the participants experienced their vision symptom, and that vision symptoms were ignored by health care personnel. They perceived a lack of information, support and acknowledgement of the impact and consequences of VIs. In contrast to physical symptoms, the participants experienced that VIs were little known symptoms, signs or sequela after stroke in health care services. VIs are difficult to observe because often they are not visible in the way an arm paresis or face palsy are. In addition, the participants also struggled to become aware, understand and acknowledge their VIs, such as when the brain fills in the missing visual field with something thought to be sensible:

“There are areas where I don’t’ see anything, but the brain makes up an image. Initially, I did not understand it, but I understand it better now. “Moving objects appear very suddenly. And, when I sit and look out – I don’t see “nothing” –- there is no area that is invisible, because there is an image there. However, if something comes in from the side, *then* I realize there is something wrong [with my visual field].”

A particular concern was that although VIs as a consequence of stroke were unknown to them, the participants also experienced that this was unknown to many health care professionals. They felt they met a lack of knowledge, competence and limited interest in their challenges, and the importance of vision in performing everyday activities. They believed this had affected their treatment from the point when they first experienced a vision symptom, in the hospital, during rehabilitation, and in long-term follow-up in the municipality. They also highlighted the concern of dealing with this alone and felt uncertain of whether the perceived improvement was something they imagined, or if it actually could be measured. Many highlighted VIs as the most important factor hindering a return to their previous life and activities. Some expressed that, if VIs had been a more focused symptom and sequela after stroke, they could probably have received appropriate vision care, which ultimately might have improved their outcomes after stroke.

#### Vision problems are experienced as a difficult unknown symptom of stroke

Several of the participants experienced a lack of awareness and knowledge, both among themselves and in the health services, of VIs as a symptom of stroke and that VIs are common after stroke. This sub-theme was identified by two categories (Table 3).

### Experience of sudden vision problems- distressful but not alarming

Five participants experienced sudden vision problems at the onset of their stroke. These participants expressed that sudden vision problems were confusing and a distressful experience. The symptoms varied greatly from minor feelings of eye irritations to double vision or severe visual field loss. A few experienced a feeling of something irritating their eye, which they tried to rub away.

“It happened while I was driving, and suddenly I thought I had something in my eye, and I started to rub … . To make it go away. I stopped the car and continued rubbing, but it did not go away.”

Another participant said he suddenly discovered that he could only read the right-hand side of the signs in a food market. He recalled that he had felt something slightly wrong with his vision the previous few hours but gave it no further attention:

“And.. I drove to Oslo … Did not feel any problems driving either. However when I got into the food market and was going to read the posters –then I got a surprise –I could not see the whole words! I could only see half the words!”

Even though he reported the experience as serious and distressful, he drove a hundred kilometers back home. Another participant explained:

“And I fell asleep [watching television]. When I woke up, half the screen was missing. There was a line up and down, and I could not see to the right. It was grey, so I called my husband and told him there was something wrong with the television. Then, I started to look around, and I noticed I could see half of everything. I rubbed my eyes … but I thought it [visual loss] would pass—I did not think it could be something serious! But this was in the evening, so I took my medication, because I got tired, so I took them and went to bed.

Another, with acute double vision experienced an overwhelming feeling of dizziness and sickness, to the point where she felt that all she could do was close her eyes and go to bed. She did not call for medical help until the next day as the symptoms persisted:

“I also lost balance, you know. It was not only that I did not see-that I saw double, but I was swaying. Just like a drunk man; I was swaying. That was unsettling. Very distressful. So, I went to bed. My son asked if we should call the doctor, but I said: “No, I will try to get some sleep”

Although the specific symptoms were new and experienced as distressful, they confused them with minor irritations or other symptoms of being unwell and hesitated to contact medical help. None recognized it as a sign of acute stroke in need of urgent medical attention:

Three participants were admitted to the hospital because of physical limb weakness, and two had their stroke as a complication of surgery and were already in hospital at stroke onset. For some, it took a while before they became aware of their VIs, and others first realized their VIs when the visual field was tested. One was unaware of something wrong until it was explained after a while by the hospital personnel that he only ate from one side of the plate:

“So … you can say, my vision, in fact, I became aware of it when I was going to eat. Then I—well, **I** had not noticed it, but the ones who served the food did: ‘Why didn’t you finish your food?’ I did not see any food on the left side of the plate.”

Participants admitted to the hospital in a hurry due to severe physical symptoms explained that they struggle to recall their first awareness of their visual problems. These were lost among other physical and more known problems.

#### Difficulties relating vision problems as a symptom of stroke

The five participants who experienced VIs as their presenting symptom of stroke, hesitated to contact medical help, as they thought it would go away. When the symptoms persisted, from hours to days, they all contacted their general practitioner (GP) and not the emergency medical services (EMS), nor were they admitted directly to the hospital but instead were called into the GP’s office. After the initial assessment, the GPs all referred the patients to the hospital, but with varying degrees of urgency. Some were admitted to hospital directly from the GPs office by ambulance or taxi, while others were sent home and asked to call the ophthalmology department in the hospital and make an appointment themselves:

“And then … (one day after) I called the hospital, and then I said, “I feel like I’m going blind!” And the woman that answered said that I should talk to the ophthalmologist. “He is occupied for a moment, but he will call you when he is available.” And he called shortly after, and I explained the same for him. “If I am covering the right eye I see like that, and if I am covering the left eye it is just the same.” “Then, it is not the eye,” he said. “You don’t have the same error on both eyes, completely the same. So, it has to be something else, and the first thing that crosses my mind is stroke,” he said. “You need to come immediately.””

One participant, who worked as a janitor, was told by a colleague that he needed to check his vision because he seemed to neglect one side of the room (eg, when clearing a room or setting up chairs). The GP checked his visual acuity and concluded everything was fine, missing his visual field loss. However, when his right arm lost its strength a bit later, he was rapidly admitted to a stroke unit.

During their stay in the stroke unit, some participants said they were informed that time was crucial for a successful outcome of medical treatment. These participants emphasized this knowledge contrasted with their actual experiences from the treatment they received. They expressed a feeling of missed opportunities for medical treatment due to time lost in the GPs’ or the ophthalmologists’ waiting rooms. One participant explains:

“I was told; “You have lost your vision. You will be blind for the rest of your life.” If I had come directly to the hospital they could have saved it [vision], this should have been within 90 minutes, but it took almost three hours before I was there, waiting”

#### Experiences of inadequate visual care

The second subtheme represents two categories that describe the participants’ experiences of visual care, rehabilitation and discharge from the stroke unit. Although they recount the overall treatment in the stroke unit as good and safe, they had other experiences of the attention, information, care and follow up regarding their VIs.

### “They primarily do not have it in their checklist” - an experienced lack of focus on vision in the stroke units

The participants described a lack of focus, awareness, and attention on their VIs in the stroke unit. They described being tested comprehensively, however, they felt focus was on their physical functions. Especially participants for whom VIs were their only or main sequela after stroke reacted to this. They described a health care system lacking competence, assessment, and follow-up of VIs after stroke:

“Yeah, they—maybe they primarily do not have it in their checklist. Because it should be just as important to check your vision as it is to check if your arm works or not! You know, or if you struggle to empty your bladder. Because they are very concerned about that. The residual urine, they call it. They check that all the time - that you have managed to empty your bladder. But if your eyes work? Not at all! It is not even on their list! That astonishes me because your eyes are pretty important. You cannot replace them with new ones. You can get new teeth, you can actually do a lot with new limbs, but you cannot do anything with your eyes.”

One became aware of her VI when her husband brought her the newspaper and she was not able to read it. She reacted very negatively to the fact that she discovered it herself:

“I asked for my book, but I could not read a word. Nothing! Then, my husband arrived and asked me to try to read the newspaper. He held it up for me and I could not understand a thing. I had to tell the doctor during the ward round that my vision was impaired. “Oh?” That was a big surprise to them.”

Another example interpreted by the participants as a lack of focus on VIs was the information regarding driving post stroke. One said he was told he would be given a driving ban for 3 months, but they forgot and he started to drive when he was discharged despite his visual field loss. Another said he was surprised the hospital did not take time to talk to him about driving, but his wife who is a nurse, stopped him from driving.

Several participants used the word “lucky” when they described their VIs after stroke, as they had seen how serious stroke outcomes could be. Others were called “lucky” by the hospital staff who compared them to others who sat in wheelchairs or had aphasia. The participants emphasized this as particularly negative and condescending, as it made them feel that their impairment was insignificant compared to others. One said:

“‘After all, you have been lucky,’ they told me. ‘It could have been much worse.’ I tried to tell them that ‘lucky’ was the wrong word. I *have* been unlucky. Of course, I see that others are very unfortunate and need help with everything. But no, I have *not* been lucky.”

### No offer of visual rehabilitation in health services - a worry

Five of the participants reported an early discharge from the hospital: apart from their VIs, they were cognitively and physically functioning well, and were told the hospital had no more to offer them. They felt worried and disappointed about the lack of information, support, and plans for visual rehabilitation when being discharged. One participant with homonymous hemianopia as his only impairment said:

“The hospital stay was probably ok, but you get discharged home. At the hospital, I asked and I asked, “What is happening with me now [with vision rehabilitation]?” “No, we do not have any to offer you,” they replied. Everyone at the hospital, the neurologist, and everyone. So there, they had nothing to offer.”

After the participant arrived at home he made an appointment with his GP. He continued:

“He [the GP] was a bit surprised. “So, you were just sent home from the hospital?” he said. “Yes,” I answered. “They told me it was the municipality’s responsibility to follow me up now.” The hospital had control over the medication and so on, but visual rehabilitation—nothing to offer.”

The participants’ were concerned of the lack of information and rehabilitation of VIs compared to physical problems after stroke. One participant expressed:

“And, if the result of my stroke had been a problem with my arm or my leg or something like that, then I would be offered rehabilitation. At the hospital. But, for vision impairments, rehabilitation is completely missing!”

### Visual impairments—big impact now and in the future

The VIs had a big impact on the participants life’s, and told that adapting to a life with VIs was a process that took time and effort. They missed professional guidance and information in this process. A few were still searching options for vision rehabilitation. One participant finally found a rehabilitation service he thought helped him, while others said they had accepted that nothing could be done for them. Many felt their vision had improved, even though they had been told there were small chances of improvements. Many participants therefore wondered if their visual recovery could have improved further with visual rehabilitation. Three categories describe their experiences and views of life 3 months post stroke and for the future.

### Difficult and exhausting to adapt to changes in everyday activities

Reading was something the participants emphasized as an important and essential part of daily living. Therefore, not being able to read was very difficult for them. They became aware of how important reading was, not only recreational reading but also to access information in newspapers, read letters, and use the internet, particularly online banking, independently. Many lost interest in watching television, as the subtexts changed too fast and the focus and energy needed to read made them tired and fatigued. Several felt their reading gradually improved, and they used different approaches to improve their reading, such as using a pen to mark the start of the sentence or reading material written in high contrast or columns, like newspaper texts. Others said they had cancelled their newspapers and stopped watching TV:

“I am going to end my newspaper subscription, because I really struggle to … .Before, I could see both sides in the paper but now I have to sit like this [moves his finger through the text] Exhausting! Very exhausting, actually. To sit and watch TV is very exhausting.

The participants said that things they normally would have managed to do right away were delayed because of their reading problems. The visual problems was a threat to independent living; and the problems caused stress like in the following utterance:

“..sometimes when things turn bad, I really just want to take a pill and be done with it. Not having to struggle with everything … for example, paying the bills. It feels hopeless. I have tried for a longer period, but I can’t get the numbers right, and I now have an online bank and I need to find it and get my son to help me.”

The participants now needed more help from their family and friends. They said it was easier to accept and ask for help with practical issues, but admitted it was much more difficult with intimate or personal issues, such as needing their children to come when visiting their GP or administering their finances. They felt this was intruding on their privacy and independence, and several expressed they did not want their children to know their concerns, have access to their health or finances.

Several of the participants noticed that they were more tired and fatigued than they were before the stroke. They wondered if the VIs made them tired because they needed to concentrate and pay much more attention when doing activities. In addition, some said that, when tired, they could not compensate as well for their VIs and their symptoms got worse.

### Life moves on, without driving

In many ways, the participants were satisfied with their lives. They had started to adjust to their “new life,” and many emphasized things they were still able to do. However, not being able to drive was something they all agreed limited their life after stroke. All except one had to cease driving temporarily due to their VIs and, at the time of the interview, were waiting for fitness-to-drive assessments. Being told they were banned from driving was dramatic, and they really hoped this would not be a permanent situation. At the same time, some reflected that they would never drive again:

“Yeah, no. I think I will be fine. I hope that I can be so good that I can pick up driving again, but at the same time, I might have to realize that I need to manage without a driver’s license too.”

The one participant who received visual rehabilitation emphasized the hope of improving his vision enough to drive was the main motivation for training. He said he hoped the effort would pay off at the fitness-to-drive assessment. Many felt that their vision had improved enough for driving safely, but they were informed after the initial perimetry that they probably would not be allowed to drive again. All experienced the driving regulations for visual function as strict:

“I feel that I could have been driving. Because if I move my head a bit I see … yeah..almost everything. But,, with this test [perimetri], when you do it, you have your chin on the chinrest and one eye patched. The tester apparently can see my pupil. If I move my head a bit she says: “Keep fixating straight ahead!””

Several said that ceasing to drive was the most significant loss after their stroke. The driving ban was especially problematic for those who did not have a driving partner or spouse. Public transportation was not something they were very familiar with, and they had to become familiar with schedules and ticket procedures, which were new to them and on digital platforms.

### Being told that nothing could be done for the vision impairment - accepted, but not convinced …

All participants experienced, to varying degrees, poor information and a lack of awareness and knowledge of their VIs from health care personnel. When the VIs were detected, they were told that the chance of recovery was small:

“The first he said to me when I came to him [ophthalmologist] was “You have had a blood clot. You will be blind [in one eye] as long as you live,” he said. “So, I can never be able to have my vision back?” I said. “Only if you believe in miracles,” he said. “Then, you may have your vision back.” It had to be a miracle if I was going to get my vision back, but he said that that would never happened.”

Some frequently repeated that they had been told that nothing could be done for their vision and accepted this. Even when some vison rehabilitation later had been suggested, the participants repeated that they initially had been informed that their vision could not be treated. One participant said:

“I already got the answer to my question. Don’t have anything to ask about. I am told that I am going to stay blind, so what can I ask about then?”

All participants experienced that their visual function had improved since the acute phase. However, it was unclear for them if this was a result of an actual improvement or if they had simply been adapting to their new situation and were now able to compensate for their VIs. One said:

“So I don’t think it has changed much. Other than that I … I guess I use my eyes in a different way. To … see»

The participants reported that their GPs knowledge and competence of VIs after stroke were poor. The feeling of getting better contrasted markedly with the information they received in the hospital, where they were told that nothing could be done. It made them concerned. They described several self-adapted compensation strategies that helped them feel improvement in everyday life and questioned whether the care services should have offered structured rehabilitation to promote further and more efficient improvement. For example, scanning the room for objects that might be in their missing visual field, seating themselves with the missing field toward the background, or inventing strategies to avoid knocking over objects, such as a cup of coffee, when grasping.

The participants missed visual rehabilitation and support to alleviate their visual loss. They experienced a lack of understanding and inappropriate solutions among municipal health care professionals, who only focused on what they felt was irrelevant low-vision aids, such as magnifying glasses or audiobooks. They felt it was their individual responsibility to identify and organize their visual care. One spent a great amount of time with his caregivers searching for visual rehabilitation services. When he finally learned that he could have been referred directly from the hospital, he was very disappointed that this had not been done:

“I was a bit resigned at first, when I felt that … I did not get help anywhere. I did not get any response. Because, I read online and found out that it was really important to get going as soon as possible when you have (vision) problems like I do, with some sort of rehabilitation. But I felt I was banging my head against the door.”

## Discussion

This study describe stroke survivors` experience of vision symptoms, health care services, and for the first time, impact of visual impairments in the first 3 months post stroke. In this study, the participants expressed that VIs after stroke received little attention throughout health care services. This had negative consequences for acute treatment, rehabilitation, and their experiences of living with VIs. The overall theme expressed by the participants in this study is that the nature of VIs post stroke often is invisible. The concept of invisible problems after stroke has traditionally been understood as cognitive or psychological problems [41, 42]. The participants talked about their VIs in the same way that other invisible impairments have been expressed [19]. Invisible problems are, by their nature, particularly challenging due to not being acknowledged and addressed by health care services, caregivers, and the persons themselves [41, 43]. Other studies have also found that VIs after stroke are a “hidden disability” which makes it harder for stroke survivors to comprehend the nature and extent of their vision loss and its ramifications [20]. A more “visible” handicap would be easier to identify and address by health care professionals [19]. Regarding vision, this is a problem because there is a lack of knowledge, competence, structured assessments, and attention among health care personnel [33, 44]. This leads to VIs not being detected and adequately cared for, leaving the individual with feelings of uncertainty and having to deal with this alone.

### Visual problems as a presenting symptom of stroke

Many participants reported that hospitalization was delayed because their presenting symptom of stroke was visual and they did not relate it to stroke. This is similar to experiences described by stroke survivors in the UK [19]. Visual symptoms have been reported as the least known symptom of acute stroke [45, 46]. Time from symptom to hospital treatment is crucial for stroke outcomes [47]. The most important factor of pre-hospital delay in acute stroke care is that patients hesitate to contact EMS [48]. To raise awareness in the general public, many campaigns have been initiated to inform about common symptoms of stroke [49, 50]. Most of these campaigns exclude visual symptoms of stroke and emphasize face palsy, limb weakness, and aphasia, with the mnemonic FAST (Face, Arm, Speech, Time) [49]. In Norway, the present campaign is Talk, Smile, and Lift [50]. The current mnemonics that focus on limb weakness, face palsy, and aphasia ignore vision symptoms as a sign of stroke. When a person presents symptoms of stroke to any health care professional, the proper intervention is admission to the hospital for acute treatment [26]. Results from this study indicate this is not the case, because many experienced they were first called in to their GPs office for and only later admitted to hospital. In the hospital, they learned that time from stroke onset to treatment is crucial, and the visual outcome might have been better if they were treated earlier. Our study supports that there is a need for non-acute medical services (such as GPs and their staff) to gain knowledge that VIs can be a symptom of stroke and to improve their competence and routines for securing an adequate response when being confronted with stroke symptoms. The five participants that had VIs as their presenting symptoms of stroke all called their GPs, which delayed their treatment and potential outcome. This is similar to a study showing that 60.7% of stroke patients who contacted their GPs were first examined in the GPs’ clinics, while of those who contacted EMS 93.7% were directly admitted to hospital for acute assessment and treatment [51]. Another study found that thrombolysis treatment rates were lower for patients with posterior circulation infarction compared to those with anterior circulation infarction, and propose this may be due to posterior circulation symptoms, such as VIs, being less stroke specific and causing delayed hospital admission [52].

Failing to identify their VIs as symptoms of stroke may have caused a delay in hospital admission, and some participants believed they might have missed the opportunity for acute treatment to recanalize (remove blood cloth) their cerebral circulation. Therefore, there is a need for acute VI to be highlighted as an acute condition, and EMS should be contacted immediately [53]. Adding visual symptoms and balance (balance and eyes, BE-FAST) to the stroke awareness campaigns may detect a greater amount of strokes from posterior circulations [53].

### Need for greater awareness of visual impairments in stroke care

The participants experienced that their VIs received little attention by the hospital staff. This is not very surprising and has been supported by other studies. A Norwegian study of persons with hemianopia after cerebral infarction found that only 9.6% were referred to visual field testing (perimetry) and only 2.3% to visual rehabilitation [31]. A lack of awareness and structure of visual assessment after stroke is also described in studies from the United Kingdom and United States [34, 54].

In this study, several of the participants asked the stroke unit staff about rehabilitation, information, and follow-up of their VIs. They felt that this was not granted. What they felt to be sparse and poor information was frustrating, and while some accepted it, some searched for information and rehabilitation elsewhere. The Norwegian guidelines for treatment and rehabilitation of stroke do not include a specific care pathway for identification and follow-up of patients with VIs but are general in their recommendations [26]. Such care pathways should be designed to secure predictable and equal visual care after stroke [54].

Some participants were told that nothing could be done to help improve their vision. This statement is true in the sense that there is no sufficient evidence to support that visual field defects can be restored with rehabilitation [24]. However, appropriate glasses, vision rehabilitation, and personalized information and guidance can improve visual function and adaption to VIs [22, 24, 34]. Valuable tools and advice on strategies to help adapt to and cope with their VIs were unavailable for participants, and they had the impression that nothing could help them. Acute stroke units, ophthalmology departments, and rehabilitation services treating stroke survivors need to gain knowledge in these processes and offer good visual assessment, provide information about the nature of the impairment, and offer proper rehabilitation.

### Reducing the impact of visual impairment on everyday life

VIs, with their limitations on daily activities, such as reading, watching television, and mobilization, were highlighted as being the most important factor limiting patients from regaining normal life after stroke. VIs after stroke are associated with reduced quality of life, decreased participation in everyday and meaningful activities, and increased depression [17, 55]. VIs also increase the risk of falls, loss of independence, and social isolation [14, 56, 57]. In addition to optimizing vision and providing sound vision advice, an assessment of the stroke survivors’ housing and mobility training may help reduce this [58, 59]. These are important aspects to consider when planning health care services.

The participants in this study reported that driving cessation was the one activity that had negatively affected their lives the most after stroke. VIs are the major reason the majority of pre-stroke drivers cease driving after stroke [60, 61]. Being banned from driving or being unable to drive is associated with reduced community integration and affects leisure activities, personal freedom, identity, and personal roles [62]. For many, it is the most important loss after their stroke [13]. These concerns should be taken seriously, and interventions to increase inside and outside mobility should be considered [13, 58].

Sudden VIs are a shock in life and leave the person with uncertainty and many questions about their future [18]. The participants in this study were uncertain of the prognosis of their Vis and whether there was anything they could have done or should do to improve their situation. They had learned that other impairments benefit from early and frequent training and wondered if that also is the case with VIs. Contact with peers through user organizations, information to caregivers or learning and coping centers, and visual rehabilitation may help these stroke survivors adapt to and cope with the situation [19, 34]. In this study, the participants felt they received poor information about their VIs and services that could possibly help them. That specific information of individual’s VIs is significant to promote understanding of their impairment has also been raised by others [19, 20]. This stresses the importance of providing the individual with specific information, even if there is limited expectation of improved measurable function. In addition to giving new insights into the needs and experiences from the participants themselves on the acute treatment, rehabilitation and living with VIs in the early months post stroke, our results elucidates findings from other studies [19, 31, 34]. The transition from specialist health care to community services and home represents a vulnerable period that needs attention to promote better outcomes after stroke [63]. Although our participants described their experiences with the Norwegian health service, similar descriptions internationally [19, 20, 34], suggests that these challenges are common among stroke survivors. Further, the results are recognisable and transferable to other countries and need to be addressed internationally.

Health care services for stroke survivors should provide personalized information and a clinical pathway for rehabilitation and follow-up of VIs [54]. The perspectives of the participants in this study contribute to new knowledge that is included in a multidisciplinary knowledge translation project to illuminate and include the experiences of stroke survivors in designing a vision-in-stroke competence-building course for community and specialist health care services in Norway.

### Limitations

The content for this research was the lived experiences of a small number of Norwegian stroke survivors with VIs living at home, and caution must be taken to generalize to all stroke survivors. Although Norwegian health care services may differ in many ways from other countries, similarities with studies outside Norway suggests that the results are transferable internationally. Most participants in this study had visual field loss, which may be due to identifying participants with the NIHSS. One limitation of NIHSS is that it does not identify central vision loss, vertical eye movement defects or perceptual problems other than neglect, which are frequent VIs after stroke [5]. This was the only tool assessing vision used in the stroke units at the time of the study, and it may be that the descriptions do not fully represent experiences with other types of VIs. We only had access to the participants own experience of VIs and other than fulfilling the inclusion criteria for participation, we do not any objective assessments of VIs performed in the health services. Although interesting, it would not add significant insight to the participants own experiences of the attention to VIs in the health services, and how VIs affect everyday life. The participants experiences of care and follow up in health services are their own, and what the health care services actually provided can differ from their experiences. However, stroke survivors are in a situation were repeated information at different times may be necessary, something our results supports. Our findings support and elucidate other qualitative and quantitative studies, and confirm public reports and statements from user organizations about the attention of VIs and possibilities of follow up after stroke.

## Conclusion

VIs are experienced as dramatic and have a significant effect on everyday life after stroke, and there is a lack of competence in and attention to VIs in acute stroke care and rehabilitation. To contribute to better acute treatment, greater awareness of VIs as a presenting symptom of stroke is needed among all health care personnel, including nonacute health care services, along with education for the general public. Our study supports that adding visual symptoms to stroke awareness campaigns is important. Furthermore, it is necessary to include structured assessments and follow-up of VIs in stroke care. The participants expressed feelings of struggling alone with an unclear vision of their future. To improve self-efficacy, acceptance, and adapting to a life with VIs, knowledge is important. This stresses the importance of providing personalized information about individual VIs, what this entails for everyday activities now and in the future, and the options for vision rehabilitation. With information and attention from competent personnel together with standardized evidence-based clinical pathways, stroke survivors with VIs can be helped in their process of adapting to their life with VIs.

## Data Availability

Due to individual privacy law, data sharing is not possible and are not publicly available. An anonymised version of the data is available upon reasonable request from the corresponding author.

## Abbreviations

VIs: Visual impairments
RCT: Randomized controlled trial
NIHSS: National Institute of Health Stroke Scale
GP: General practitioner
EMS: Emergency medical services
FAST: FACE Arm Speech Time
